# An Opportunity During Antenatal Services to Strengthen Nurturing Care: Global and National Recommendations for Routine Ultrasound Before 24 Weeks Gestation

**DOI:** 10.3389/fpubh.2020.589870

**Published:** 2021-01-08

**Authors:** Wiedaad Slemming, Roisin Drysdale, Linda M. Richter

**Affiliations:** ^1^Department of Paediatrics and Child Health, Faculty of Health Sciences, University of the Witwatersrand, Johannesburg, South Africa; ^2^DSI-NRF Centre of Excellence in Human Development, University of the Witwatersrand, Johannesburg, South Africa

**Keywords:** antenatal, ultrasound, development, pregnancy, fetal, parenting

## Abstract

**Introduction:** The *Healthy Pregnancy, Healthy Baby* study (HPHB) augments a routine service (pregnancy ultrasound) with information about fetal and infant development and the importance of parent wellbeing and infant care, to assess whether it will improve child development and growth, parent-infant attachment, parental wellbeing and routine clinic attendance. This paper outlines the process of intervention development and implementation in a complex environment with multiple stakeholders.

**Methods:** Study participants were recruited from pregnant women attending fetal ultrasound (US) at Chris Hani Baragwanath Hospital (CHBH), Soweto, South Africa. Partners were invited to attend all sessions. The HPHB intervention, a novel combination of a health and a parenting intervention that augments a routine service (US), is being tested through a randomized controlled trial with outcome assessments at 6 weeks and 6 months follow-up. The current study outlines the process of moving from intervention design to full implementation in a high-risk clinical setting.

**Results:** Formative research informed the design and content of the intervention materials. Implementation is monitored through weekly reports and team meetings as well as formal and informal feedback received from staff and participants. Close collaborations with clinicians enhanced recruitment practices and provided clinical oversight of the trial procedures. Ongoing stakeholder engagement informed intervention procedures and strategies to address challenges that arise during implementation.

**Conclusion:** This study emphasizes the importance of dynamic, inclusive and interactive approaches to intervention development and implementation, as well as the purposeful use of varied information from diverse sources in decision-making for effective implementation.

## Introduction

The 2017 Lancet Series *Advancing Early Childhood Development: From Science to Scale*, made two critical recommendations for scale-up of early childhood development (ECD) interventions. First, reaffirming a life course approach to promoting and supporting ECD and reiterating the importance of preconception health and wellbeing of mothers and the critical first 1,000 days of life. Second, that the health sector, which has frequent contacts with pregnant women and young children, provides an ideal entry point for scaling up interventions for ECD (1). Effective interventions that promote ECD, such as skin-to-skin contact, breastfeeding and micronutrients for mothers and infants, are already delivered through the health sector and these can be built on and expanded feasibly and affordably to include additional benefits (1, 2).

The 2015 South African Department of Health Guidelines for Maternity Care recommend that all pregnant women attending a district hospital should receive one basic obstetric US at 18–20 weeks gestation to determine intra-uterine pregnancy, fetal viability, multiple births and gestational age (3). Further, the country has adopted the 2016 World Health Organization (WHO) Antenatal Care Guidelines, which recommends eight antenatal visits and one US before 24 weeks gestation (4).

This provides a highly receptive context for integrated interventions that render multiple benefits without adding costs to current vertically delivered services. Beyond the health and survival benefits of early US, evidence suggests that US could promote early attendance and use of Antenatal Care (ANC) services (5). Pregnancy US is a highly formative experience and provides an opportunity for promotive activities, such as breastfeeding counseling during pregnancy, as breastfeeding intentions are usually established by the third trimester (6, 7). To capitalize on the potential added benefits, the *Healthy Pregnancy Healthy Baby* intervention is a novel combination of a health and a parenting intervention that augments a routine service (US) with information about fetal and infant development and the importance of parent wellbeing and infant care.

**Figure 1:**
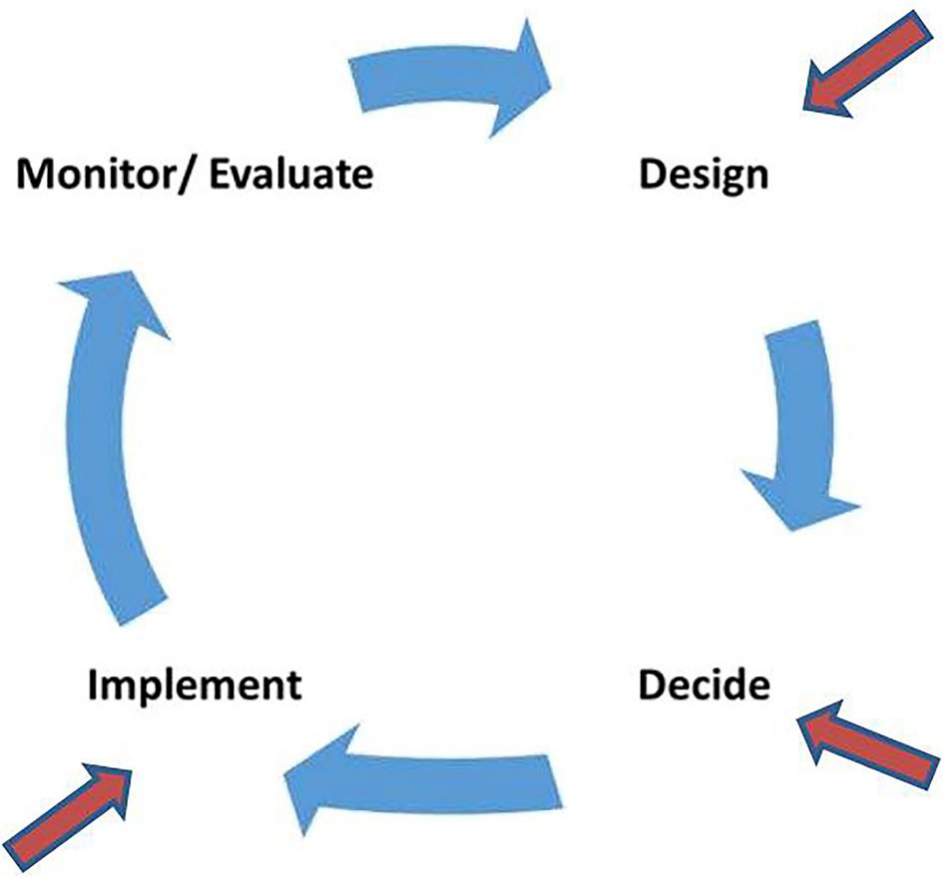
Reflections on specific stages of the implementation process.

The aim is to assess whether this intervention will improve child development and growth, breastfeeding practices, mother/father-infant attachment, maternal and paternal wellbeing and routine clinic attendance. This paper reflects on our experiences of implementing a novel intervention in a complex environment with multiple key stakeholders. The paper focuses on the initial stages of the implementation process (Figure 1), viz. design (conceptualization and intervention development), decide (reflection on decision-making at key stages to illustrate our process) and implement (adaptation and initial steps of implementation).

## Materials and Methods

### Study Design and Setting

The HPHB study is a randomized controlled trial, which started recruiting from February 2018, with ongoing follow-up.

Study participants were recruited from women attending ANC at Chris Hani Baragwanath Hospital (CHBH), a tertiary level teaching hospital, in Soweto, Johannesburg. Women, with pregnancy risk factors such as hypertension, diabetes and HIV, among others, are referred to CHBH from community health centers. After screening for fetal risk by the Fetal Medicine Unit (FMU), recruited participants attended US visits at the SAMRC/Wits Developmental Pathways for Health Research Unit (DPHRU), located at CHBH.

### Study Participants

Two hundred and forty-nine participants were recruited from pregnant women screened at CHBH. Eligibility criteria included age 18 years and older, residence within Soweto, a singleton pregnancy and <25 weeks gestation. Women were excluded if referral to CHBH indicated major fetal abnormalities and/or severe maternal morbidities, or if a woman was attending specialist antenatal clinics. Eligibility screening occurred in two stages. First, age, place of residence and gestational age of the woman was recorded to alert the FMU team that a woman was potentially eligible for study inclusion. Second, the FMU screened women for clinical risk factors, which determined whether a woman was able to be recruited into the study. If fetal abnormalities were detected during US, women were excluded from our trial and followed up at specialist clinics.

### Study Procedures

#### Formative Research and Stakeholder Engagement

Study procedures were informed through formative research comprising evidence review and qualitative research conducted with pregnant women attending ANC at CHBH prior to the trial inception. Focus group discussions and in-depth interviews were conducted with women during pregnancy and approximately 6 weeks after birth to explore their expectations and experiences of pregnancy, as well as how nurturing care can be promoted through the health system (8).

Multilevel stakeholder engagement strategies were employed during intervention conceptualization and development, and continuously throughout implementation of the trial. Stakeholder engagement included meetings/workshops and presentations with policy makers, researchers/academics, clinicians and FMU staff, civil society organizations, programme managers, and representatives from public benefit and multilateral organizations working in the field.

#### Intervention Procedures

Eligible women were randomized to one of three study arms. Arm 1, the control group, received one US at DPHRU <25 weeks gestation, where standard fetal growth measurements were taken but no intervention was delivered. Arm 2 received the study intervention at the first research ultrasound visit. Arm 3 received the intervention at two ultrasound visits' at <25 weeks and <36 weeks gestation. They received the same intervention as Arm 2. All study US were additional to those received as part of routine care at CHBH. Partner involvement and interest in the pregnancy and baby were encouraged by inviting men to attend the study US and follow-up visits in all arms.

**Figure 2:**
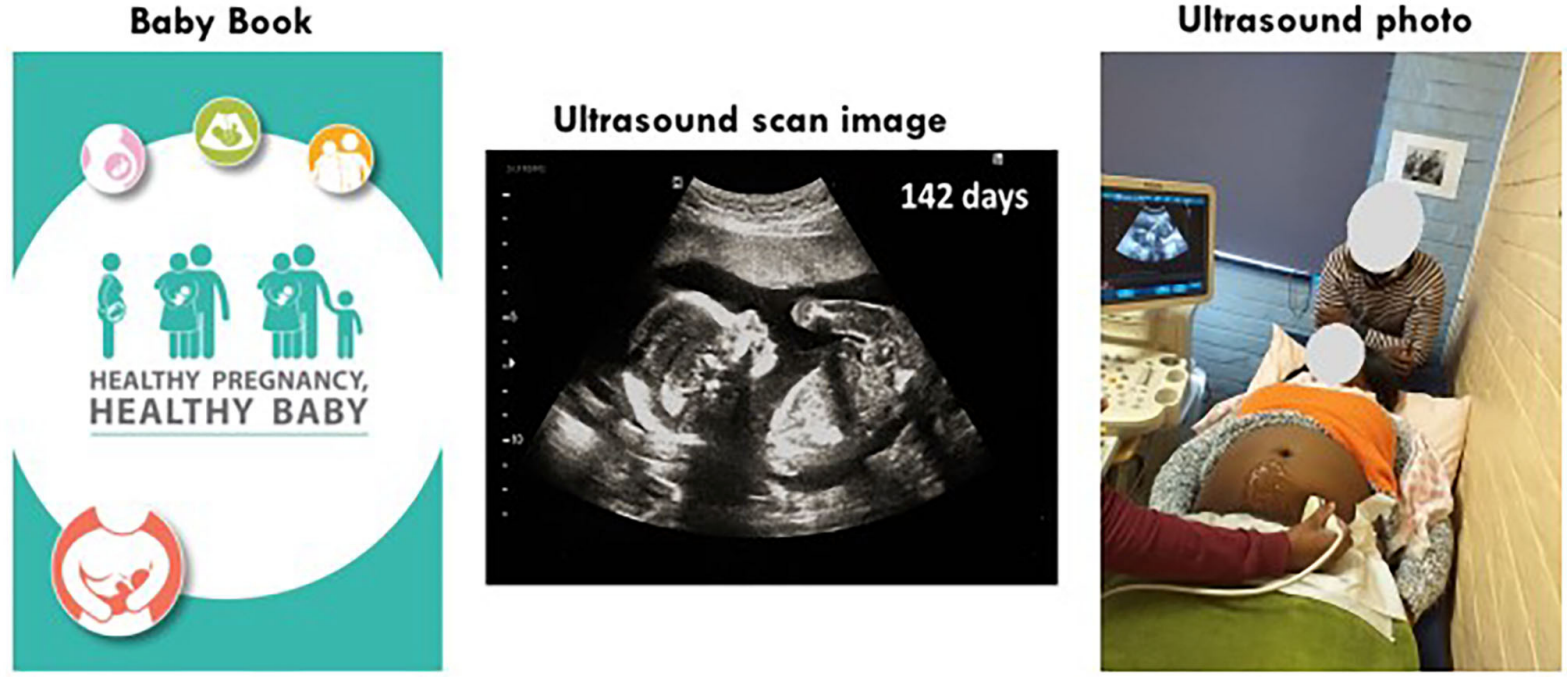
Intervention components.

Mothers in the intervention arms received a small baby book with information related to fetal and infant development, maternal wellbeing, partner support, breastfeeding and parenting preparedness. Participants in the intervention arms also received hardcopies and digital ultrasound images and a photograph of the woman having the US, with her partner, where present. Digital images were sent to participants via WhatsApp to share with family and friends. Printed images were given to participants during the session, which could be attached to the baby book (Figure 2).

The intervention was delivered by trained sonographers who, due to the nature of the intervention, were not blinded to arm allocation. Study sonographers underwent 2-day training to standardize ultrasound procedures and another 1-day refresher training conducted by a senior sonographer and educator. As the focus of the intervention was new to sonographers, additional training (by the study investigators) was provided on how to communicate about fetal development with participants (and their partners) and how to deliver and reinforce messages and information about maternal wellbeing and child development using the study resource materials.

**Figure 3:**
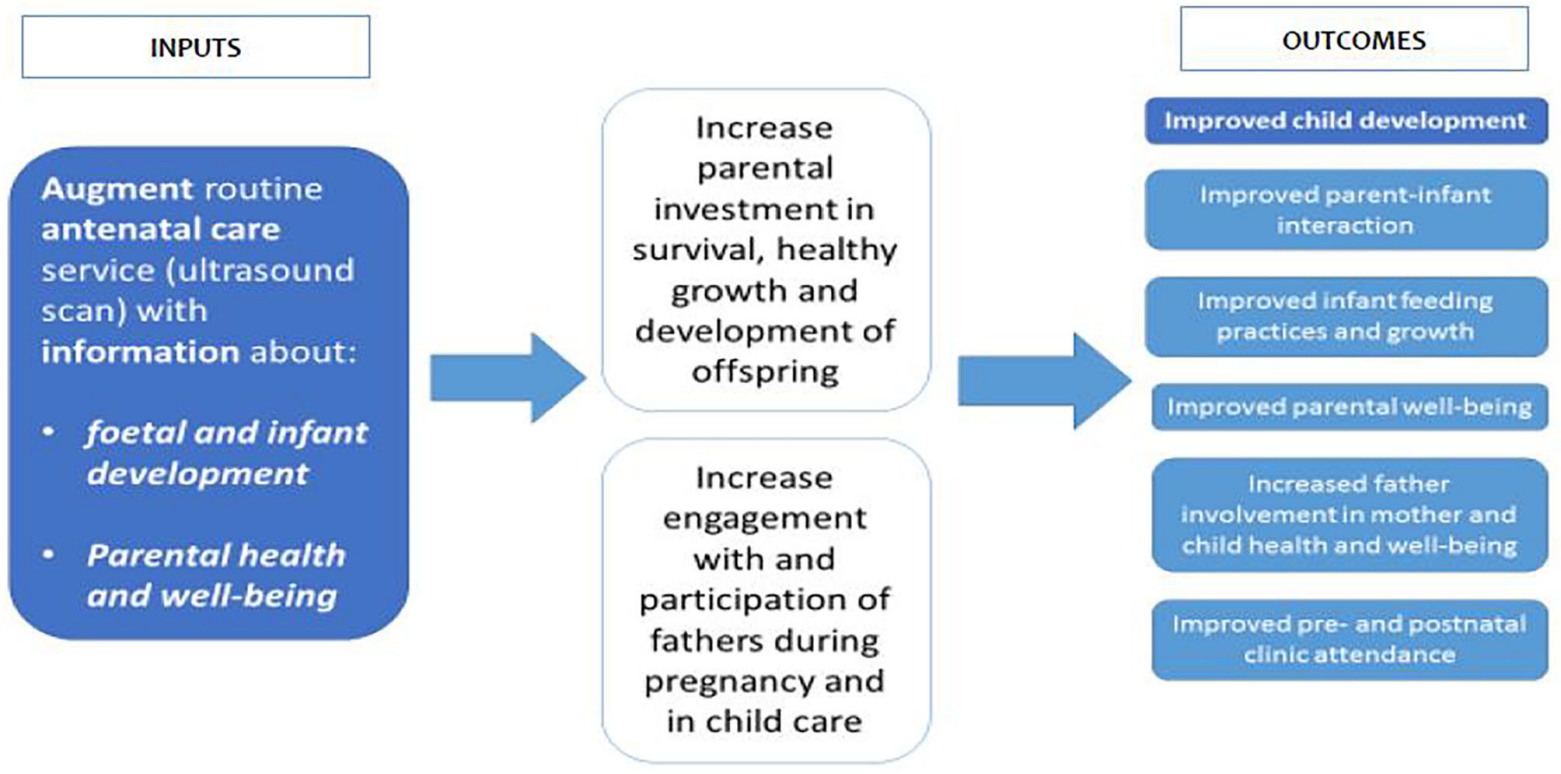
Implementation framework for the *Healthy Pregnancy, Healthy Baby* study.

Figure 3 shows the implementation framework for the study. Further information on the study procedures are available in the trial protocol (9).

### Data Collection and Management

Data are collected on the enrolled women, their partners (where available) and the infant by trained independent assessors at three stages, viz. (1) baseline at the first study US visit at DPHRU <25 weeks gestation; (2) 6-week postnatal follow-up; and (3) 6-month outcome assessment.

Baseline data collection included information on the participant's sociodemographic information, reproductive history and feeding intentions. The participant and her partner completed a social support questionnaire and an US experience questionnaire after the session. Maternal and Paternal Antenatal Attachment Scales (10, 11) were used to assess fetal attachment and the Edinburgh Postnatal Depression Scale (12) to identify parents at risk of depression.

Six-week follow up questionnaires and measures were completed with mothers, infants and partners by trained assessors. Questionnaires included information on the perinatal and postnatal period, breastfeeding practices, infant behavior (sleep, crying etc.), and partner and social support. Postnatal depression was assessed for the mother and partner using the Edinburgh Postnatal Depression Scale. Infant weight and length were measured following standard procedures, using the SECA 367 infant scale for weight and SECA 416 infantometer for length. Anthropometric measures were converted to *z*-scores using the 2006 WHO Growth Standards for LAZ, WAZ, and WLZ. Birth and postnatal information, including on maternal and infant birth outcomes, were collected from the official child health record.

Study outcomes are assessed at 6-month follow-up by trained assessors who are blind to the arm allocation using (1) questionnaires administered by interview, (2) direct child assessments, and (3) videotaped mother-infant interactions.

#### Primary Outcome

Infant cognitive, language/communication, motor and social-emotional development is assessed at infant age 6 months using the Bayley Scales of Infant and Toddler Development (Bayley-III), which has been validated in SA (13, 14). Assessments are conducted by occupational therapists and physiotherapists trained in child development and blinded to participant allocation. The Home Screening Questionnaire (HSQ), a 30-item parent-report tool, is used to identify features of the home environment related to childhood development (15). The HSQ has been validated and used for research purposes in SA and does not require a home visit (16, 17).

#### Secondary Outcomes

Mother-infant interaction is rated to agreement, by two blind assessors, from a 5-min videotaped observation of mothers and infants “talking and playing with each other.” The infant is placed in a highchair, with the mother seated at the same height opposite the infant and they have an attractive stack toy to share. An adapted coding scheme developed by Richter et al. (18), is used to code engagement (eye contact, joint attention), emotional tone, emotional regulation (comforting for distress), responsiveness and scaffolding.

Infant weight and length measures, the Edinburgh Postnatal Depression Scale and interview-administered questionnaires on breastfeeding practices, partner and social support are repeated. Information on infant immunization status and clinic attendance are obtained from the child's health record (Road to Health Book).

#### Intervention Fidelity

Participant adherence is assessed by counts (e.g., visit attendance) and intervention delivery was monitored by observing contacts between the sonographer and participants to record the nature of interactions and assess the quality of intervention delivery and data collected. Intervention dose was measured by recording duration of ultrasound visits (with and without delivery of the intervention). Participant experience was monitored by gathering participant and partner views on the ultrasound experience.

To ensure quality of study procedures, all staff were trained on the principles of research ethics and Good Clinical Practice, including informed consent and how to communicate with participants with low literacy levels. They were also trained on how to complete the study questionnaires, appropriate referral procedures, study operating procedures and how to manage sensitive issues and difficult situations. Outcome assessors are Masters-level occupational- and physiotherapists with a minimum of 5 years' experience in conducting child development assessments. They were trained to administer the child development outcome assessments, as well as to set up the assessment space, establish rapport with infants, and accurate recording etc.

Study data are collected and managed using REDCap (Research Electronic Data Capture), which is a secure, web-based application designed to support research. REDCap enhances data quality by checking for missing data, providing audit trails for tracking data management and export procedures, controlling questions that must be completed for each participant, and detecting invalid answers in real time (19). Standard operating procedures (SOPs) support the delivery of the study procedures and intervention according to the trial protocol. Research staff received thorough training, standardization and close monitoring of adherence to data collection and management procedures. Data are checked regularly by the study coordinator and data manager to ensure quality.

This paper describes the process of intervention development, adaptation and implementation, outlining the decision-making processes and influences at particular stages of the implementation process.

### Ethics

The Human Research Ethics Committee (Medical) of the University of the Witwatersrand, South Africa, provided ethical approval for the study (M181915). The trial is registered with https://pactr.samrc.ac.za (PACTR201808107241133).

## Results

### Description of the Intervention Development, Adaptation, and Implementation

#### Intervention Design and Development

##### Formative Research

Existing evidence indicate that women value seeing their baby and hearing the fetal heartbeat via ultrasound and find the experience emotional and reassuring (5, 20–22). Similar findings have been reported from research conducted with fathers, and the presence of the father appeared to have a positive effect on their pregnant partner (23). US provides an opportunity to engage fathers to promote support for their partner during pregnancy and active involvement in child care (24). There was also evidence showing that there is a demand for pregnancy US, with some women opting to pay for additional scans if needed (25). The additional benefits US may offer, beyond survival-focused clinical care, reinforced the decision to try to leverage this existing service to promote ECD starting in pregnancy, and to engage fathers.

Qualitative findings reaffirmed that women wanted to see the fetal image, did not know they could hear the heartbeat and were often told little other than their expected date of delivery and whether or not there were complications or abnormalities. Many women indicated that they would have liked more information and to be able to share the experience with others (8). These findings confirmed that the US interaction is currently underutilized as an opportunity to engage pregnant women and their partners in the development and wellbeing of their unborn baby and in preparation for parenting. It provided the study team with a better understanding of the current landscape in which practices were embedded and the perceptions, motivations and priorities of the intended target audience (pregnant women and their partners), to inform intervention development and implementation. It also confirmed the importance of including partners in the intervention and the need for information on their role during pregnancy and parenting.

##### Intervention Delivery and Content Development

Most women attending ANC at CHBH are referred from community health centers with general risk factors. CHBH is one of the world's biggest hospitals and attends to ~24,000 births annually. This meant that the research team had to work closely with the clinicians and staff at the unit to refine screening criteria and recruitment procedures to minimize disruptions to services. The FMU clinicians also helped to define the referral procedures when abnormalities were detected at study US and to tailor the overall intervention design and components to better fit the complex implementation environment.

The next step in the process was to develop the detail of intervention components and materials. This was achieved through workshops with research staff to review formative research findings, discussions with key stakeholders (programme managers, policy makers etc.) and consultations with the clinical staff at the CHBH ANC unit. These were supported by the concurrent development of study SOPs, protocols and tools. One of the main decisions that arose from these engagements is that the intervention had to be “light touch,” in order to be delivered feasibly by an existing health workforce (i.e., sonographers) without significant additional investments, role expansion and burden to already strained services.

Stakeholder engagement workshops held early in the design phase were useful for aligning the interventions with current health system priorities and ensured that policy makers and those responsible for potential scaling up felt the intervention was relevant and feasible. Shifting from a “survive” to “survive and thrive” orientation within the health sector, South Africa's child health record, the Road to Health Book (RTHB), has been redesigned. There is also a move to defining a “first 1,000-day service package” and this led to decisions to align the “look and feel” of the intervention content and material to the RTHB. As current national pregnancy resources are not oriented in the same way nor aligned with the RTHB, this provided the opportunity to test materials that could influence practice if proven beneficial.

Pretesting intervention content and materials allowed us to identify and adapt components that were misunderstood, did not adequately communicate intended messages or were not relevant to our target audience. Pretesting of the intervention materials was conducted via focus group discussions with pregnant women from Soweto. The sessions were led by an experienced, multilingual qualitative researcher, with an observer present, and elicited feedback on comprehension, acceptability and relevance of the content and materials, used to refine final versions.

#### Recruitment and Retention Strategies

The complex health environment and additional screening requirements posed specific challenges to recruitment as there was a select pool of women from which to sample. The screening US was conducted by FMU staff within a busy public health service, which resulted in slow recruitment. Strategies to overcome recruitment difficulties that did not over-burden clinical staff were agreed upon through regular face-to-face meetings and liaison with FMU staff. For example, recruiters assisted sonographers in completing the screening checklists with women at US during busy periods. We also adopted more proactive recruitment strategies, such as increasing the number of recruiters on-site to follow up all potentially eligible participants before and after ANC consultation and their FMU ultrasound.

Every enrolled participant received a partner invitation card, which contained a brief outline of the study and invited partners to attend the study US. However, partner participation in the study was lower than anticipated. In order to address this, with the agreement of participants, the study team contacted partners shortly after enrollment and made follow-up phone calls closer to the study visit to encourage attendance. Weekend sessions were included to accommodate those partners who worked during the week. The participant and her partner were provided with a photograph of them attending the US session to enable discussions about the experience and to elicit interest and possible future partner participation. Research staff make regular phone and WhatsApp contact with participants and their partners in-between study visits to promote retention in the study. We also follow-up participants who default to encourage and facilitate future follow up attendance, and with those who withdraw to understand their reasons for dropping out of the trial. Follow-up interviews are conducted by telephone for partners who are unable to attend physically.

#### Continuous Review and Adaptation

We designed a dashboard to monitor study indicators and reports are submitted by research staff on the required indicators and discussed with the study team on a weekly basis to review and address issues/concerns. Staff found the original study dashboard and weekly progress reports confusing, which led to adaptations in the design to simplify the reporting process. Management of the overall dashboard became the responsibility of a data manager and reporting of the weekly numbers was the responsibility of the project coordinator. These changes, as well as regular data and implementation monitoring meetings involving the whole research team, assist with data quality control. It ensures that any issues (such as incorrect data capture) are dealt with promptly, and any required adaptations to data collection or management processes are made timeously. It also facilitates self- and team data quality monitoring.

It became evident that the intervention depends largely on the interpersonal skills of the sonographer to deliver the messages effectively. This information was gained from observational data collected using a variety of sources, i.e., data collected on participant experiences of the US, observations by a senior researcher, reflections from research staff and self-reflection by the sonographer. In this way, an interactive and inclusive approach to data collection (and use) was able to highlight specific issues so that necessary adaptations (in this case, increased supervision and support) could be made timely and collaboratively.

The research staff observed that partners who attended the US visit, displayed great interest in the encounter and were often very emotional during the session when they visualized their baby and heard the fetal heartbeat. Participants who attended with their partners also shared messages with the study team about how the experience had positively influenced their partner's support and interest in the pregnancy, and in their wellbeing and that of the baby. Due to these qualitative observations and the informal feedback received, we submitted an ethics amendment to videotape a small number of couples during the US to illustrate, especially men's responses to the encounter.

#### Collaboration and Networks

We established a broad stakeholder network at the start of the trial to allow for engagement and input into the design, adaptation and implementation processes. It also served to keep key stakeholders informed of progress and lessons learnt during the course of the study. These engagement processes created as a valuable platform to problem solve challenges, garner support for the research and explore how the lessons learnt and any beneficial findings could be translated into practice. There is considerable interest in the trial, due to the novelty and intuitive appeal of the intervention to stakeholders, who perceive the intervention as having a potential triple benefit for children: survival, health and development.

At an operational level, close collaboration with the clinical management and staff of FMU were critical from the start to provide clinical input and oversight of the trial procedures. This allowed for more effective planning and review of intervention design and implementation, including the recruitment and training of study sonographers, refining study recruitment procedures and defining clear referral pathways.

## Discussion

This paper reports on the HPHB study processes used to advance from intervention development, adaptation and implementation, and outlines the decision-making influences and procedures at different phases of the implementation process. The study highlights the need for dynamic approaches to intervention design, adaptation and implementation, the importance of using different types of information from a variety of sources for decision-making and the essential role of inclusive and interactive approaches for intervention development and implementation.

Due to the highly receptive context for potential scale-up of the augmented US innovation through the health system, it was important to include considerations of feasibility, replicability and scalability into designing our intervention from the start. We wanted to maximize this opportunity to integrate an ECD intervention into existing health services rather than try to deliver and scale up a new intervention with additional human resource requirements. Hence, it was important to be responsive to challenges and opportunities that arose during intervention design and implementation to improve the prospects of translating the augmented US intervention into routine practice, if shown to be beneficial.

Adopting a dynamic approach to implementation enabled the research team to make the necessary adaptations to design and delivery and to be sensitized to and leverage opportunities to enhance implementation. Using formal and informal data from our study participants and research staff have helped to complement routine data collection methods, such as including videos of fathers attending the US, as well as guide our decision-making throughout implementation. This is beneficial for the study at this time but will also enable better acceptance and translation of interventions when moving to scale (26, 27).

Regular, “whole team” data monitoring procedures allowed for better data quality (due to improved peer- and self-monitoring), and responsivity when needs for adaptation and improvement arose in response to lessons learnt. Collecting and using quantitative and qualitative data from a variety of sources helped the study team to continuously reflect and monitor implementation progress and quality. These data sources include data about US experiences, observational data collected during sessions, WhatsApp and SMS messages sent by participants about their US experiences, video clips of the sessions, weekly monitoring meetings and reports and regular team debriefing and feedback about progress. Often more traditional forms of feedback, such as data monitoring reports are more valued with less attention paid to the need for, and contribution of, interaction platforms for group and personal reflection as part of the implementation monitoring process (28). Dedicating time for regular reflection by research staff, as well providing opportunities for interactions (formal and informal) that allow participants to share their experiences and thoughts, helped to promote shared learning and continuous improvements along the way.

The formative research confirmed assumptions made by the study team and influenced the nature and delivery of the intervention. Collaborations with the CHBH clinical services and broader stakeholder engagement also played an important role in aligning the research question with local and national priorities, learning from implementation processes and challenges, and providing greater transparency in study procedures and decision-making. This is highlighted as important in intervention research (29, 30), and was also found to be essential here.

Areas that require strengthening include gaining a better understanding of how to address issues faced with recruitment and retention of partners. Despite employing a number of strategies, this remains a challenge throughout the study and requires a more partner-centered approach moving forward. Using interactive and inclusive approaches to data collection is a strength of this study but also an area that should be built on when transitioning to scale. Focused strategies to include more qualitative data, using a variety of methods, would enhance the interpretation of quantitative findings.

Health systems have a number of “moving parts” that are inter-related and interactive (31, 32). We have had to consider these interdependencies and allow for self- and collective reflection on the implementation process and procedures using inclusive approaches (involving all stakeholders) in our decision making in order to adapt and be flexible.

It is clear that successful scaling strategies require dynamic, inclusive and interactive approaches to intervention development and implementation, as well as the purposeful use of varied information from a range of sources for decision-making. Reflecting on the study implementation processes has provided us with a better understanding of the strengths and weaknesses related to our implementation strategy and monitoring and evaluation system. This allows us to work on ways to address deficiencies and build on strengths moving forward but also contributes to the evidence-base on how to address implementation challenges in complex settings, especially when planning for scale-up.

## Data Availability Statement

The data supporting the conclusions of this article will be made available by the authors upon reasonable request.

## Ethics Statement

The studies involving human participants were reviewed and approved by Human Research Ethics Committee (Medical), University of the Witwatersrand, South Africa. The patients/participants provided their written informed consent to participate in this study.

## Author Contributions

WS wrote the paper. LR and RD made substantial contributions and approved the final version for publication. All authors read and approved the final manuscript.

## Conflict of Interest

The authors declare that the research was conducted in the absence of any commercial or financial relationships that could be construed as a potential conflict of interest.
